# Use of Patient-Reported Outcomes Measurement Information System Measures in Clinical Research in Patients With Stroke: A Systematic Literature Review

**DOI:** 10.1016/j.arrct.2022.100191

**Published:** 2022-03-25

**Authors:** Henk J. Arwert, Daniella M. Oosterveer, Jan W. Schoones, Caroline B. Terwee, Thea P.M. Vliet Vlieland

**Affiliations:** a Department of Rehabilitation, Leiden University Medical Center, Leiden, the Netherlands; b Basalt Rehabilitation, Leiden, the Netherlands; c Leiden University Medical Center, Leiden, the Netherlands; d Department of Epidemiology and Data Science, Amsterdam University Medical Center, Amsterdam, the Netherlands; e Department of Orthopedics, Rehabilitation, and Physical Therapy, Leiden University Medical Center, Leiden, the Netherlands

**Keywords:** Patient reported outcome measures, Rehabilitation, Stroke, Systematic review, GH, Global Health, ICF, International Classification of Functioning, Disability, and Health, ICHOM, International Consortium for Health Outcomes Measurement, mRS, modified Rankin scale, PROMIS, Patient-Reported Outcomes Measurement Information System

## Abstract

**Objective:**

To systematically describe the use and outcomes of Patient-Reported Outcomes Measurement Information System (PROMIS) measures in clinical studies in populations with stroke.

**Data Sources:**

A systematic search on the use of PROMIS measures in clinical stroke studies in 9 electronic databases.

**Study Selection:**

Studies had to be original, reporting on outcome data using PROMIS measures in populations with stroke (ischemic and/or hemorrhagic), from January 1st, 2007. Initially, 174 unique studies met the inclusion criteria. In 2 steps, titles, abstracts and full-text articles were screened for eligibility (2 authors independently).

**Data Extraction:**

From the selected articles, study characteristics, type of PROMIS measures, and its outcomes were extracted by 2 authors independently. The authors discussed their views to achieve consensus. A third author was consulted if necessary.

**Data Synthesis:**

In total, 27 studies (24,366 patients) were included, predominantly from the United States (22); most study populations were hospital-based (20); the number of patients ranged from 30-3283. In general, patients had no or mild symptoms (median modified Rankin scale 1). Two different generic PROMIS measures were reported (PROMIS Global Health, PROMIS 29) and 9 PROMIS measures focusing on specific domains (sleep, pain, physical functioning, self-efficacy, satisfaction with social roles, depression, anxiety, cognition, fatigue). These match the International Classification of Functioning, Disability, and Health (ICF) domains mentioned in the Core Set for Stroke. The measures were administered 1-55 months after stroke. Outcome data are provided. Pooling of data was not achieved because of a large variety in study characteristics (inclusion criteria, follow-up moments, data processing).

**Conclusions:**

The PROMIS measures in this review could be relevant from a patient's perspective, covering ICF core set domains for patients with stroke. The large variety in study characteristics hampers comparisons across populations. Many different outcome measures are used to report results of stroke rehabilitation studies.

The development of the International Classification of Functioning, Disability, and Handicap (ICF) Core Set for Stroke was initiated in 2004 to reach general agreement on the scope of concepts to measure and on the instruments to be used in stroke disability, describing the problems patients with stroke can be confronted with in terms of functioning, activities, and participation.^1^ Also in 2004 the development of the Patient-Reported Outcomes Measurement Information System (PROMIS) started, sponsored by the US National Institutes of Health in an effort to address major concerns about the status of patient-centered outcome measures because the widely used legacy instruments are limited by a lack of precision, standardization, and comparability of scores across studies and diseases.^2^^,^^3^ In 2007 the PROMIS became available.^4^

The next step, the development of an international consensus how to uniformly report on the health outcomes after stroke, was made in 2016 by the International Consortium for Health Outcomes Measurement (ICHOM).^5^ This resulted in the Stroke Standard Set (https://ichom.org/files/medical-conditions/stroke/stroke-reference-guide.pdf), describing how and when to report on initial conditions, risk factors, and outcomes in a standardized manner. This opens up new possibilities to compare performance of health care globally, allow clinicians to learn from each other, and improve the care provided to stroke patients.

PROMIS measures are item-response theory-based questionnaires that cover generic as well as specific domains of health that are relevant for many (patient) populations. All PROMIS measures use a standardized metric, centered on the United States population. The use of a normalized distribution (T score 0-100; standardized mean, 50±10) enhances interpretability.

PROMIS measures have been applied in general populations and in people with various physical conditions (spinal surgery, critical illness, low back pain, cancer at a young age, chronic pain, during rehabilitation).^6^, ^7^, ^8^, ^9^, ^10^, ^11^ This also holds for populations with stroke where several PROMIS measures are used since its introduction. Specifically, the PROMIS Global Health (GH) is relevant in this respect. ICHOM promotes the use of PROMIS GH as part of routine outcome measurement for patients with stroke at 90 days follow up.^5^ The PROMIS GH consists of 10 items summarized into 2 component scores: a Global Physical Health score and a Global Mental Health score. It offers reliable and precise measures of generic symptoms and quality of life.^12^ For the PROMIS GH the psychometric properties were evaluated in a recent systematic review, reporting evidence for a sufficient internal consistency, reliability, and validity in populations with stroke.^13^ The aim of this review was to systematically describe the use and outcomes of PROMIS measures in clinical studies in populations with stroke, in particular the PROMIS GH.

## Methods

### Search strategy

The search was performed by a trained librarian in 9 electronic databases (PubMed, MEDLINE [OVID version], Embase [OVID version], EmCare, PsycINFO [EbscoHOST version], Google Scholar, Academic Search Premier, Web of Science, Cochrane Library). Stroke and PROMIS Medical Subject Headings of the National Library of Medicine terms and free-text words were used. The search period started in January 1, 2007, because PROMIS became available in that year. The search was performed on August 14, 2020, and updated on April 12, 2021. Full details of the search strategy can be found in the appendix.

### Selection criteria

Inclusion criteria: (1) studies reporting on outcome data using 1 or more PROMIS measures, (2) including patients with stroke (ischemic and/or hemorrhagic) aged 18 years or older, and (3) written in English, French, German, or Dutch.

Exclusion criteria: studies including exclusively patients with transient ischemic attacks or subarachnoid hemorrhages because these patients have a distinct clinical course. Only original studies were included. No further limitations were formulated on the type of study design (eg, retrospective studies, prospective studies, randomized controlled trials). If patient groups with stroke and other medical conditions were included, information on patients with stroke had to be reported separately. The reference lists of systematic literature reviews obtained from the search were used to identify potentially eligible clinical studies on the subject (snowballing method or backward reference tracking).

### Selection process

The selection of studies was systematically done by 2 authors independently, using Rayyan Systems Inc.^14^ Screening of the records concerned reading title and abstract using the abovementioned eligibility criteria; subsequently, the full texts of resultant studies were screened, using the same inclusion and exclusion criteria. In case of disagreement the 2 authors discussed their views to achieve consensus. If agreement was not reached a third author was consulted.

### Data extraction

A data extraction form was used to systematically extract information from the full-text articles. The data extraction was done by 1 of the authors, with all results checked by a second author.

Regarding the study characteristics, information on the first author, year of publication, country, and study design (cross-sectional, cohort, trial, other; based on definition of the original authors) was retrieved. With respect to the study populations the following information was retrieved: follow-up post stroke (time points), setting (hospital-based, community-based, other), the number of patients, general patient characteristics (mean age, sex), stroke type and location, neurovascular interventions, modified Rankin scale (mRS) (yes/no) score. The mRS score assesses disability in patients with stroke, with a score ranging from 0-6, with 0 meaning no symptoms and 6 meaning death.^15^ Concerning the PROMIS measures used, the name of the measures were recorded and the actual results were extracted.

## Results

**Fig 1:**
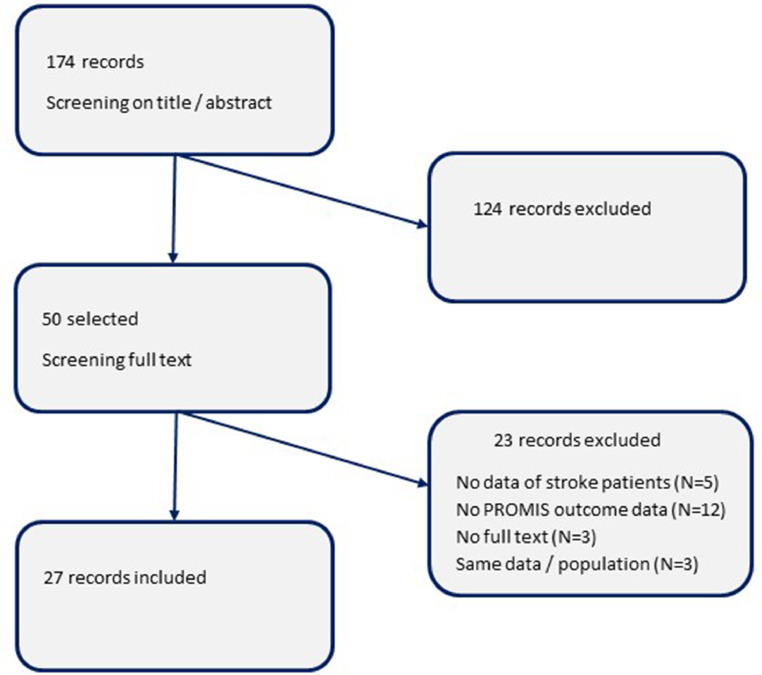
Flowchart of records reporting on PROMIS in populations with stroke.

The systematic search resulted in 174 unique records. The screening of titles and abstracts resulted in the exclusion of 124 records, of which 15 were systematic literature reviews. Snowballing revealed no additional studies to include. During screening of the 50 full-text articles that were retrieved, another 23 studies were excluded (fig 1). Three of these excluded studies reported on the psychometric properties of specific PROMIS measures (validity, responsiveness) but were excluded because actual outcome data were not reported.

The characteristics of the included 27 studies (24,366 patients in total) are summarized in table 1.^16^, ^17^, ^18^, ^19^, ^20^, ^21^, ^22^, ^23^, ^24^, ^25^, ^26^, ^27^, ^28^, ^29^, ^30^, ^31^, ^32^, ^33^, ^34^, ^35^, ^36^, ^37^, ^38^, ^39^, ^40^, ^41^, ^42^ Studies were predominantly from the United States origin (22 of 27; 81%). The majority of the studies concerned hospital-based populations with stroke, describing the outcomes of a prospective or retrospective outpatient cohort according to data gathered in regular care, at regular poststroke care visits (20 of 27; 74%). Three studies used the same population of survivors of stroke selected for a randomized controlled trial; the outcomes of the control group and the intervention group were combined.^20^^,^^25^^,^^35^ Duration of follow-up was given in 24 studies, varying from 1-55 months.

**Table 1:** Characteristics of clinical studies in patients with stroke using PROMIS measures

Author	Country	1 Cross-sectional 2 Cohort 3 RCT/Open Trial	Follow-up (mo)	1 Hosp 2 Comm 9 Other	N	Mean Age ± SD	Female (%)	Isch (%)	Affected Side % R/L/O	mRS	PROMIS GH	Physical Function	Fatigue	Pain Interference	Anxiety	Sleep Disturbance	Satisfaction With Social Roles	Depression	PROMIS 29	Cognitive Function	Self-efficacy
Naidech et al^16^	US	2	12	1	149	NA	NA	0		x		x									
Katzan et al^17^	US	2	2.5	1	1946	63.1±14.2	46.1			x		x									
Katzan et al^18^	US	2	2.6	1	2431	62.9±14.4	46.4			x		x	x	x‖	x	x	x				
Katzan et al^19^	US	2	6.5	1	3283	63.5±14.4	46			x		x	x								
Chen et al^20^	US	3	3	9	258	61.7±10.8	19							x							
Katzan et al^21^	US	2	3.3	1	1195	62±15	45.1			x		x	x	x	x	x	x				
Katzan et al22⁎	US	2	4.7	1	1407	61.5±14.8	44.9	100		x		x	x	x	x	x	x				
Lam et al^23^	NL	1	12	1	75	68.9±11.2	32	100	31/40/29		x										
Rose et al^24^	US	1		3	1359	80.7±6.8	52												x		
Chen et al25†	US	3	3	9	258	61.7±10.8	19							x							
Katzan et al^26^	US	2	1	1	496	61.2±15.9	45.8	86.1		x		x	x	x			x			x	
Lapin et al^27^	US	2	3.5	1	1351	60.5±14.9	45.1			x	x										
Lapin et al28†	US	2	6	1	337	61±14	55.8			x		x	x		x		x				
Reeves et al29†	CND	3	3	1	265	66.2±13.2	49	86		x	x										
Shulman et al^30^	US	1	55	1	166	55.6±13.2	66.4			x	x	x	x		x			x			x
Byun et al^31^	US	1		1	100	60±12.7	50	85								x§					
Hreha et al^32^	US	1		9	182	69.4±2.9	40.7												x		
Katzan et al33*	US	2	3.2	1	1412	60.6±14.9	44.8	100		x	x		x			x					
Katzan et al34*	US	2	2.2	1	2190	60.5±14.9	44.9	100		x		x	x	x	x		x				
Kroenke et al^35^	US	3	3	9	258	61.7±10.8	19											x			
Ogunlade et al^36^	US	1	7	3	450	61.7±11.1	44											x			
Rhudy et al37†	US	2	6	1	30	55.6±9.4	30	100	37/53/10				x					x		x	
Graaf et al^38^	NL	2	3	1	360	71 (17)‡	39.7	93	54/46/-	x	x										
Katzan et al^39^	US	2	4.3	1	1696	62.9±14.6	48.8	62.7		x	x	x	x	x	x	x	x				
Lapin et al^40^	US	1	2.5	9	200	62.2±13.3	41.5	81		x	x	x	x	x	x	x	x				
Lens et al^41^	B	1	3	1	102	NA	NA												x		
Rimmele et al^42^	GER	2	3	1	482	71.9±12.88	48.5	100			x										

Abbreviations: B, Belgium; CND, Canada; Comm, community-based; GER, Germany; Hosp, hospital-based; Isch, ischemic; NL, the Netherlands; mRS, modified Rankin scale; RCT, randomized controlled trial; US, United States of America.

⁎ Only ischemic stroke subgroup.

† Data available at baseline and follow-up.

‡ Median (IQR).

§ PROMIS Sleep Disturbance and PROMIS Sleep-Related Impairment.

‖ PROMIS Pain Intensity.

Relevant stroke characteristics were not available in a majority of the studies; 3 studies described which side of the brain was affected.^23^^,^^37^ None of the studies reported on neurovascular interventions such as intra-arterial thrombolysis of thrombectomy, nor on related complications. The use of the mRS score was reported in 17 of 27 studies (63%). In 14 of those 17 studies, the reported median mRS score was 1 (recorded at time points ranging from 1-55 months after stroke), indicating “no significant disability despite symptoms.” In 1 study the outcome of the PROMIS Physical Function was stratified per mRS score.^16^ In 2 studies the mRS score was provided as a mean outcome, varying from 1.3-1.7 points.^29^^,^^30^

In total, 11 PROMIS measures were identified in this review. Twelve studies used a PROMIS measure for Physical Function, 12 used a PROMIS Fatigue measure, 9 used a PROMIS Pain measure, and 9 used the PROMIS GH. The PROMIS measure for Anxiety and Satisfaction With Social Roles and Activities were used in 8 studies, and the PROMIS Sleep Disturbance and/or Sleep-Related Impairment were used in 7. PROMIS Depression was used in 4 studies, and the PROMIS 29 in as used in 3. The PROMIS 29 entails the following domains: Depression, Anxiety, Physical Function, Pain Interference, Fatigue, Sleep Disturbance, and Ability to Participate in Social Roles and Activities, plus 1 Pain Intensity question (0-10 numeric rating scale); The reported outcomes were component scores or scores per domain. PROMIS Cognitive Function was used twice, PROMIS Self-efficacy for Managing Chronic Conditions (managing daily activities, symptoms, medications and treatments, emotions, social interactions) was used once. The details of the PROMIS measures are provided in table 2, and the PROMIS outcomes are shown in table 3.

**Table 2:** PROMIS item bank as stated by the original authors

Author	PROMIS Item Bank
Naidech et al^16^	Physical Function
Katzan et al^17^	Physical Function 1.0 (CAT)
Katzan et al^18^	Physical Function (CAT). Satisfaction With Social Roles and Activities (CAT). Fatigue (CAT). Anxiety (CAT). Pain Interference (CAT). Sleep Disturbance (CAT).
Katzan et al^19^	Physical Function 1.0. Fatigue 1.0
Chen et al^20^	Pain Interference; 6-item (Short Form), 4-item, 6-item, 8-item
Katzan et al^21^	Physical Function (CAT). Satisfaction With Social Roles and Activities (CAT). Fatigue (CAT). Anxiety (CAT). Pain Interference (CAT). Sleep Disturbance (CAT).
Katzan et al^22^	Physical Function (CAT). Satisfaction With Social Roles and Activities (CAT). Fatigue (CAT). Anxiety (CAT). Pain Interference (CAT). Sleep Disturbance (CAT).
Lam et al^23^	PROMIS GH
Rose et al^24^	PROMIS 29 2.0; 4 items each category
Chen et al^25^	Pain Interference; 6-item (Short Form), 4-item, 6-item, 8-item
Katzan et al^26^	Physical Function (CAT). Satisfaction With Social Roles and Activities (CAT). Fatigue (CAT). Cognitive Function (CAT). Pain Interference (CAT).
Lapin et al^27^	PROMIS GH
Lapin et al^28^	Physical function (CAT). Satisfaction with social roles and activities (CAT). Fatigue (CAT). Anxiety (CAT).
Reeves et al^29^	PROMIS GH
Shulman et al^30^	PROMIS GH. Depression; 8-item. Anxiety; 8-item. Fatigue; 8-item. Physical Function; 12-item. Self-efficacy for managing chronic conditions.
Byun et al^31^	Sleep Disturbance; 8-item. Sleep-Related Impairment; 8-item
Hreha et al^32^	PROMIS 29; 4 items each category
Katzan et al^33^	PROMIS GH. Sleep Disturbance 1.0. Fatigue 1.0
Katzan et al^34^	Physical Function (CAT). Satisfaction With Social Roles and Activities (CAT). Fatigue (CAT). Anxiety (CAT). Pain Interference (CAT).
Kroenke et al^35^	Depression; 8-item (Short Form), 4-item, 6-item, 8-item
Ogunlade et al^36^	Depression; 8-item (Short Form)
Rhudy et al^37^	Fatigue. Cognitive Function. Depression.
Graaf et al^38^	PROMIS GH
Katzan et al^39^	PROMIS GH. Physical Function (CAT). Satisfaction With Social Roles and Activities (CAT). Fatigue (CAT). Anxiety (CAT). Pain Interference (CAT). Sleep Disturbance (CAT).
Lapin et al^40^	PROMIS GH. Physical Function (CAT). Satisfaction With Social Roles and Activities (CAT). Fatigue (CAT). Anxiety (CAT). Pain Interference (CAT). Sleep Disturbance (CAT).
Lens et al^41^	Physical Function. Ability to Participate in Social Roles and Activities. Fatigue. Anxiety. Depression. Pain Interference. Sleep Disturbance. (PROMIS 29)
Rimmele et al^42^	PROMIS GH

**Table 3:** Outcomes of PROMIS measures in populations with stroke

Author	GH (T score), mean ± SD	Sleep (T score), mean ± SD*	Pain (T score), mean ± SD†	PF (T score), mean ± SD	SE (T score), mean ± SD	Sat Soc Role (T score), mean ± SD	Depression (T score), mean ± SD	Anxiety (T score), mean ± SD	PROMIS 29 (T score), mean ± SD	Cognition (T score), mean ± SD	Fatigue (T score), mean ± SD
Naidech et al^16^				mRS 0: 52.7±7.1 mRS 1: 46.1±6.1 mRS 2: 39.9±5.6 mRS 3: 33.9±6.5 mRS 4: 26.1±8.5 mRS 5: 17.6±4.7							
Katzan et al,^17^ median (IQR)				41.9 (33.3-49.7)							
Katzan et al18‡		49.6±10.8	53.4±10.8	40.6±11.3		43.2±11.6		52.5±10.7			53.2±10.9
Katzan et al,^19^ median (IQR)				40.9 (33.1-48.8)							52.2 (46.3-60.3
Chen et al20§			53.2±10.4‖ 53.1±10.6¶ 53.1±10.6# 53.2±10.3⁎⁎								
Katzan et al^21^		49.2±10.5	52.2±10.8	58.8±10.7††		55.4±11.3††	49.8±10.8	52.3±10.2			53.2±10.5
Katzan et al^22^		49.2±10.5	52.3±10.7	58.4±10.6††		54.8±11.4††	49.5±10.9	52.0±10.2			52.9±10.6
Lam et al^23^	GPH 45.8±9.9 GMH 49.6±9.1										
Rose et al24‡‡									PHS 42.2±9.2 MHS 50.1±8.0		
Chen et al25§§			Baseline 53±10.5 3 mo 53±10								
Katzan et al^26^			50.2±10.8	59.2±10.4††		54.8±11.3††					53.1±10.3
Lapin et al^27^	GPH 45.8±9.2‖‖ GPH 39.8±7.9¶¶ GMH 47.5±9.0‖‖ GMH 41.6±8.4¶¶										
Lapin et al^28^				Baseline 42.1±10.8 6 mo 45.1±10.5		Baseline 45.2±11.7 6 mo 48.8±11.3		Baseline 49.9±10.4 6 mo 48.6±9.6			Baseline 51.5±10.3 6 mo 49.7±9.4
Reeves et al,29## least-square means (95% CI)	GPH Baseline 42.8 (41.4-44.14) GPH 3 mo 43.1 (41.7-44.5) GMH Baseline 46.0 (44.0-47.9) GMH 3 mo 47.1 (45.2-49.1)										
Shulman et al^30^	GPH 49.7±7.3 GMH 46.1±10.4			45.2±9.7	50.8±8.9⁎⁎⁎ 52.3±9.9††† 50.4±8.4‡‡‡ 51.2±9.5§§§ 53.0±8.5‖‖‖		47.9±9.6	50.6±9.8			50.9±8.7
Byun et al^31^		56.36±6.21¶¶¶ 53.30±3.49									56.22±6.25
Hreha et al^32^			53.8±10.3	41.3±8.8			51.1±8.8	49.5±8.8	###		52.6±10.4
Katzan et al^33^	GPH 44.5±9.5 GMH 46.2±9.2	49.5±10.5									53.4±10.4
Katzan et al^34^		49.4±10.5	52.4±10.8	41.3±10.6		44.6±11.2		52.6±10.0			53.5±10.5
Kroenke et al^35^							51.3±9.2‖ 50.5±10.0¶ 50.3±9.9# 50.0±10.3⁎⁎				
Ogunlade et al^36^				41.30±10.09							
Rhudy et al,^37^ median (range)							Baseline 53.40 (38.4-68) 6 mo 48.30 (38.4-80.3)			Baseline 40.63 (23.13-63.17) 6 mo 49.95 (28.55-63.17)	Baseline 57.50 (33.4-76.8) 6 mo 51.65 (33.4-73)
Graaf et al38****											
Katzan et al^39^	GPH 44.4±9.1 GMH 46.2±9.0	49.9±10.2	52.2±10.6	41.7±10.4		45.6±11.1		52.0±10.1			52.8±10.3
Lapin et al^40^	GPH 43.4±9.0 GMH 47.0±9.0	50.2±10.2	52.3±9.8	39.6±9.6		45.5±9.8	50.4±9.0	51.4±9.0			53.7±9.5
Lens et al^41^		48.9±2.8	50.2±3.7	57.4±2.8††		51.5±2.3††	50.7±3.3	52.5±3.0	###		50.3±2.3
Rimmele et al^42^	GPH 39.9±6.31 GMH 43.5±8.77										

NOTE. For details regarding the studies listed refer to table 1.

Abbreviations: GH, PROMIS GH; GMH, Global Mental Health; GPH, Global Physical Health; MHS, Mental Health scores PF, Physical Function; PHS, Physical Health scores; SE, Self-efficacy for Managing Chronic Conditions; Sat Soc Role, Satisfaction With Social Roles and Activities.

⁎ Sleep Disturbance unless stated otherwise.

† Pain Interference unless stated otherwise.

‡ Pain Intensity.

§ Pain Interference.

‖ 4-item.

¶ 6-item.

# 8-item.

⁎⁎ Short Form.

†† Reversed scores (higher is worse).

‡‡ PROMIS 29: PHS (Physical Health summary score), MHS (Mental health summary score).

§§ Mean of 4 scales.^‖,¶,#,**^

‖‖ Self-reported.

¶¶ Proxy-reported.

## Only usual care group.

⁎⁎⁎ PROMIS SE Managing Daily Activities.

††† PROMIS SE Managing Symptoms.

‡‡‡ PROMIS SE Managing Meds/Treatments.

§§§ PROMIS SE Managing Emotions.

‖‖‖ PROMIS SE Managing Social Interactions.

¶¶¶ Sleep-related impairment.

### PROMIS 29.

⁎⁎⁎⁎ Outcome was not a T score (54.3±18.5).

Of the 9 studies reporting outcomes on the PROMIS GH, 6 did so in line with the recommendation of the ICHOM (follow-up 2.5-3.5 months), 5 of which also reported the mRS score outcome. In 1 study a total score of the PROMIS GH was calculated (instead of a normalized T score), which is inappropriate.^38^ In the other 5 studies the outcomes on Global Mental Health ranged from 43.5-47.5, and the outcomes on Global Physical Health ranged from 39.9-45.8.^29^^,^^27^^,^^33^^,^^40^^,^^42^

## Discussion

In this review 27 studies used PROMIS as outcome measure in patients with stroke, mostly published after the Stroke Standard Set was developed in 2016. Six of these reported PROMIS outcomes in line with the Stroke Standard Set (PROMIS GH at 3-month follow-up).

Apart from the PROMIS GH, as a general outcome measure of health care-related quality of life, outcome data were available on the PROMIS measures assessing Sleep Disturbance or Sleep-Related Impairment, Pain Interference of Pain Intensity, Physical Function, Self-efficacy, Satisfaction With Social Roles and Activities, Depression, Anxiety, Cognitive Function, and Fatigue. These measures cover relevant domains as described in the ICF Core Set for Stroke.^1^ Sleep, pain, physical function, depression, anxiety, cognition, and fatigue are related to the ICF dimension Body functions. Physical function also covers aspects of Activity limitations. Satisfaction With Social Roles and Self-efficacy are measures related to the dimension Participation and the Environment chapter Support and Relationships.

For PROMIS measures other than the PROMIS GH the results of the psychometric properties reliability, validity, and responsiveness are not yet reviewed. The studies in this review were too diverse in terms of inclusion criteria, follow-up moments, and data processing to summarize the psychometric properties systematically. The PROMIS data in studies found were predominantly collected at regular outpatient appointments. Patients with stroke available for outpatient follow-up probably show relatively favorable outcomes compared with patients with stroke unable to attend outpatient appointments. This is supported by the median mRS score of 1 in the majority of the included studies (if available), indicating that at least 50% of the participants experienced no significant disability despite symptoms after stroke, or no symptoms at all.

In 1 study the PROMIS outcomes were not calculated as T scores, hampering comparison.^38^ Furthermore, we noted that the PROMIS Physical Function and PROMIS Satisfaction With Social Roles and Activities were reported inconsistently, scoring from worse to good or in reverse (see table 3). Lower scores should indicate a worse outcome, and higher scores should indicated a better outcome. For interpretability it is mandatory to adhere to this standardized direction of scale.^43^

### Study limitations

The outcomes of the PROMIS measures showed a considerable variety, partly because of differences in study characteristics (design, definition of population of stroke, moment of follow-up). Therefore, a comparison of outcomes across populations or a meta-analysis is impeded.

## Conclusions

PROMIS measures are available and being used to measure domains relevant for patients with stroke. Despite the methodological advantages of PROMIS measures over classical patient-centered outcome measures they are reported infrequently in studies outside the United States. The large variety in study characteristics limits comparison across populations. The recommendation of the ICHOM to use the PROMIS GH as standard outcome measurement 3 months after stroke was followed in a limited number of studies in current stroke literature. Preferably, in future research on stroke outcomes international guidelines such as ICHOM should be followed.

## Appendix Supplementary materials

Image, application 1
