# Supplementary material for: Use of Patient-Reported Outcomes Measurement Information System Measures in Clinical Research in Patients With Stroke: A Systematic Literature Review
Source: Arch Rehabil Res Clin Transl. 2022 Mar 25;4(2):100191. doi: 10.1016/j.arrct.2022.100191 (PMC9214304; doi:10.1016/j.arrct.2022.100191)
Supplement: Supplementary file 1 [file mmc1.docx]

Supplement

*PubMed*

(("Stroke"[mesh] OR "Stroke"[tw] OR "strokes"[tw] OR "CVA"[tw] OR "CVAs"[tw] OR "Cerebrovascular Accident"[tw] OR "Cerebrovascular Accidents"[tw] OR "Cerebrovascular Insult"[tw] OR "Cerebrovascular Insults"[tw] OR "CVI"[tw] OR "CVIs"[tw] OR "Brain Attack"[tw] OR "Brain Attacks"[tw] OR Brain Infarct*[tw] OR "Brain Infarction"[mesh] OR "Cerebrovascular Apoplexy"[tw] OR "Apoplexy"[tw] OR "Brain Vascular Accident"[tw] OR "Brain Vascular Accidents"[tw] OR "Cerebrovascular Stroke"[tw] OR "Cerebrovascular Strokes"[tw] OR "Cerebral Stroke"[tw] OR "Cerebral Strokes"[tw] OR "Acute Stroke"[tw] OR "Acute Strokes"[tw] OR "Acute Cerebrovascular Accident"[tw] OR "Acute Cerebrovascular Accidents"[tw] OR "Cerebrovascular Event"[tw] OR "Cerebrovascular Events"[tw] OR "Cerebrovascular Attack"[tw] OR "Cerebrovascular Attacks"[tw] OR "Cerebral Infarction"[mesh] OR Cerebral infarct*[tw] OR "Brain ischemia"[mesh] OR "Brain ischemia"[tw] OR "Brain ischaemia"[tw] OR "Cerebral Ischemia"[tw] OR "Cerebral Ischaemia"[tw] OR "Cerebral Hemorrhage"[mesh] OR Cerebral Hemorrhag*[tw] OR Cerebral Haemorrhag*[tw] OR Intracerebral Hemorrhag*[tw] OR Brain Hemorrhag*[tw] OR Brain Haemorrhag*[tw] OR Intracerebral Haemorrhag*[tw] OR "Neurologic Event"[tw] OR "Neurologic Events"[tw] OR "Ischemic Attack, Transient"[mesh] OR "transient ischemic attack"[tw] OR "transient ischemic attack"[tw] OR "transient ischaemic attack"[tw] OR "transient ischaemic attacks"[tw] OR "TIA"[tw] OR "TIAs"[tw] OR "transient brainstem ischemia"[tw] OR "transient brainstem ischaemia"[tw] OR "transient brain stem ischemia"[tw] OR "transient brain ischemia"[tw] OR "transient brain ischaemia"[tw] OR "transient cerebral ischemia"[tw] OR "transient cerebral ischaemia"[tw] OR "transient cerebral ischemic"[tw] OR "transient cerebral ischaemic"[tw] OR ("transient"[tw] AND ("brain"[tw] OR "cerebral"[tw]) AND (ischemi*[tw] OR ischaem*[tw]))) AND ("PROMIS"[tw] OR "PROMIS10"[tw] OR "NIHPROMIS"[tw] OR "PROMISPI"[tw] OR "PROMISPF"[tw] OR "PROMISGH"[tw] OR "PROMISSF"[tw] OR "Patient Reported Outcome Measurement Information System"[tw] OR "patient reported outcome measurement system"[tw] OR "patient reported outcome measurement"[tw] OR "Patient Reported Outcomes Measurement Information System"[tw] OR "patient reported outcomes measurement system"[tw] OR "patient reported outcomes measurement"[tw] OR (("Patient-Reported Outcome*"[tw] OR "Patientreported Outcome*"[tw] OR "PROM"[tw] OR "PROMS"[tw]) AND ("Information System*"[tw] OR "measurement system*"[tw] OR "item bank*"[tw] OR "itembank*"[tw])))) AND (english[la] OR german[la] OR dutch[la] OR french[la]) AND ("2007/01/01"[PDAT] : "3000/12/31"[PDAT])

*MEDLINE*

((exp "Stroke"/ OR "Stroke".mp OR "strokes".mp OR "CVA".mp OR "CVAs".mp OR "Cerebrovascular Accident".mp OR "Cerebrovascular Accidents".mp OR "Cerebrovascular Insult".mp OR "Cerebrovascular Insults".mp OR "CVI".mp OR "CVIs".mp OR "Brain Attack".mp OR "Brain Attacks".mp OR Brain Infarct*.mp OR exp "Brain Infarction"/ OR "Cerebrovascular Apoplexy".mp OR "Apoplexy".mp OR "Brain Vascular Accident".mp OR "Brain Vascular Accidents".mp OR "Cerebrovascular Stroke".mp OR "Cerebrovascular Strokes".mp OR "Cerebral Stroke".mp OR "Cerebral Strokes".mp OR "Acute Stroke".mp OR "Acute Strokes".mp OR "Acute Cerebrovascular Accident".mp OR "Acute Cerebrovascular Accidents".mp OR "Cerebrovascular Event".mp OR "Cerebrovascular Events".mp OR "Cerebrovascular Attack".mp OR "Cerebrovascular Attacks".mp OR "Cerebral Infarction"/ OR Cerebral infarct*.mp OR exp "Brain ischemia"/ OR "Brain ischemia".mp OR "Brain ischaemia".mp OR "Cerebral Ischemia".mp OR "Cerebral Ischaemia".mp OR exp "Cerebral Hemorrhage"/ OR Cerebral Hemorrhag*.mp OR Cerebral Haemorrhag*.mp OR Intracerebral Hemorrhag*.mp OR Brain Hemorrhag*.mp OR Brain Haemorrhag*.mp OR Intracerebral Haemorrhag*.mp OR "Neurologic Event".mp OR "Neurologic Events".mp OR exp "Ischemic Attack, Transient"/ OR "transient ischemic attack".mp OR "transient ischemic attack".mp OR "transient ischaemic attack".mp OR "transient ischaemic attacks".mp OR "TIA".mp OR "TIAs".mp OR "transient brainstem ischemia".mp OR "transient brainstem ischaemia".mp OR "transient brain stem ischemia".mp OR "transient brain ischemia".mp OR "transient brain ischaemia".mp OR "transient cerebral ischemia".mp OR "transient cerebral ischaemia".mp OR "transient cerebral ischemic".mp OR "transient cerebral ischaemic".mp OR ("transient".mp AND ("brain".mp OR "cerebral".mp) AND (ischemi*.mp OR ischaem*.mp))) AND ("PROMIS".mp OR "PROMIS10".mp OR "NIHPROMIS".mp OR "PROMISPI".mp OR "PROMISPF".mp OR "PROMISGH".mp OR "PROMISSF".mp OR "Patient Reported Outcome Measurement Information System".mp OR "patient reported outcome measurement system".mp OR "patient reported outcome measurement".mp OR "Patient Reported Outcomes Measurement Information System".mp OR "patient reported outcomes measurement system".mp OR "patient reported outcomes measurement".mp OR (("Patient-Reported Outcome*".mp OR "Patientreported Outcome*".mp OR "PROM".mp OR "PROMS".mp) AND ("Information System*".mp OR "measurement system*".mp OR "item bank*".mp OR "itembank*".mp)))) AND (english.la OR german.la OR dutch.la OR french.la) AND (2007 OR 2008 OR 2009 OR 2010 OR 2011 OR 2012 OR 2013 OR 2014 OR 2015 OR 2016 OR 2017 OR 2018 OR 2019 OR 2020 OR 2021 OR 2022).yr

*Embase*

((exp "cerebrovascular accident"/ OR "Stroke".mp OR "strokes".mp OR "CVA".mp OR "CVAs".mp OR "Cerebrovascular Accident".mp OR "Cerebrovascular Accidents".mp OR "Cerebrovascular Insult".mp OR "Cerebrovascular Insults".mp OR "CVI".mp OR "CVIs".mp OR "Brain Attack".mp OR "Brain Attacks".mp OR "Brain Infarct*".mp OR exp "Brain Infarction"/ OR "Cerebrovascular Apoplexy".mp OR "Apoplexy".mp OR "Brain Vascular Accident".mp OR "Brain Vascular Accidents".mp OR "Cerebrovascular Stroke".mp OR "Cerebrovascular Strokes".mp OR "Cerebral Stroke".mp OR "Cerebral Strokes".mp OR "Acute Stroke".mp OR "Acute Strokes".mp OR "Acute Cerebrovascular Accident".mp OR "Acute Cerebrovascular Accidents".mp OR "Cerebrovascular Event".mp OR "Cerebrovascular Events".mp OR "Cerebrovascular Attack".mp OR "Cerebrovascular Attacks".mp OR "Cerebral infarct*".mp OR exp "Brain ischemia"/ OR "Brain ischemia".mp OR "Brain ischaemia".mp OR "Cerebral Ischemia".mp OR "Cerebral Ischaemia".mp OR exp "Brain Hemorrhage"/ OR "Cerebral Hemorrhag*".mp OR "Cerebral Haemorrhag*".mp OR "Intracerebral Hemorrhag*".mp OR "Brain Hemorrhag*".mp OR "Brain Haemorrhag*".mp OR "Intracerebral Haemorrhag*".mp OR "Neurologic Event".mp OR "Neurologic Events".mp OR "Transient Ischemic Attack"/ OR "transient ischemic attack".mp OR "transient ischemic attack".mp OR "transient ischaemic attack".mp OR "transient ischaemic attacks".mp OR "TIA".mp OR "TIAs".mp OR "transient brainstem ischemia".mp OR "transient brainstem ischaemia".mp OR "transient brain stem ischemia".mp OR "transient brain stem ischaemia".mp OR "transient brain ischemia".mp OR "transient brain ischaemia".mp OR "transient cerebral ischemia".mp OR "transient cerebral ischaemia".mp OR "transient cerebral ischemic".mp OR "transient cerebral ischaemic".mp OR ("transient".mp ADJ3 ("brain".mp OR "cerebral".mp) ADJ3 (ischemi*.mp OR ischaem*.mp))) AND ("PROMIS".mp OR "PROMIS10".mp OR "NIHPROMIS".mp OR "PROMISPI".mp OR "PROMISPF".mp OR "PROMISGH".mp OR "PROMISSF".mp OR "Patient Reported Outcome Measurement Information System".mp OR "patient reported outcome measurement system".mp OR "patient reported outcome measurement".mp OR "Patient Reported Outcomes Measurement Information System".mp OR "patient reported outcomes measurement system".mp OR "patient reported outcomes measurement".mp OR (("Patient-Reported Outcome*".mp OR "Patientreported Outcome*".mp OR "PROM".mp OR "PROMS".mp) AND ("Information System*".mp OR "measurement system*".mp OR "item bank*".mp OR "itembank*".mp)))) NOT (conference review or conference abstract).pt AND (english.la OR german.la OR dutch.la OR french.la) AND (2007 OR 2008 OR 2009 OR 2010 OR 2011 OR 2012 OR 2013 OR 2014 OR 2015 OR 2016 OR 2017 OR 2018 OR 2019 OR 2020 OR 2021 OR 2022).yr

*Web of Science*

(TI=("cerebrovascular accident" OR "Stroke" OR "strokes" OR "CVA" OR "CVAs" OR "Cerebrovascular Accident" OR "Cerebrovascular Accidents" OR "Cerebrovascular Insult" OR "Cerebrovascular Insults" OR "CVI" OR "CVIs" OR "Brain Attack" OR "Brain Attacks" OR "Brain Infarct*" OR "Brain Infarction" OR "Cerebrovascular Apoplexy" OR "Apoplexy" OR "Brain Vascular Accident" OR "Brain Vascular Accidents" OR "Cerebrovascular Stroke" OR "Cerebrovascular Strokes" OR "Cerebral Stroke" OR "Cerebral Strokes" OR "Acute Stroke" OR "Acute Strokes" OR "Acute Cerebrovascular Accident" OR "Acute Cerebrovascular Accidents" OR "Cerebrovascular Event" OR "Cerebrovascular Events" OR "Cerebrovascular Attack" OR "Cerebrovascular Attacks" OR "Cerebral infarct*" OR "Brain ischemia" OR "Brain ischemia" OR "Brain ischaemia" OR "Cerebral Ischemia" OR "Cerebral Ischaemia" OR "Brain Hemorrhage" OR "Cerebral Hemorrhag*" OR "Cerebral Haemorrhag*" OR "Intracerebral Hemorrhag*" OR "Brain Hemorrhag*" OR "Brain Haemorrhag*" OR "Intracerebral Haemorrhag*" OR "Neurologic Event" OR "Neurologic Events" OR "Transient Ischemic Attack" OR "transient ischemic attack" OR "transient ischemic attack" OR "transient ischaemic attack" OR "transient ischaemic attacks" OR "TIA" OR "TIAs" OR "transient brainstem ischemia" OR "transient brainstem ischaemia" OR "transient brain stem ischemia" OR "transient brain stem ischaemia" OR "transient brain ischemia" OR "transient brain ischaemia" OR "transient cerebral ischemia" OR "transient cerebral ischaemia" OR "transient cerebral ischemic" OR "transient cerebral ischaemic" OR ("transient" AND ("brain" OR "cerebral") AND (ischemi* OR ischaem*))) AND TS=("PROMIS" OR "PROMIS10" OR "NIHPROMIS" OR "PROMISPI" OR "PROMISPF" OR "PROMISGH" OR "PROMISSF" OR "Patient Reported Outcome Measurement Information System" OR "patient reported outcome measurement system" OR "patient reported outcome measurement" OR "Patient Reported Outcomes Measurement Information System" OR "patient reported outcomes measurement system" OR "patient reported outcomes measurement" OR (("Patient-Reported Outcome*" OR "Patientreported Outcome*" OR "PROM" OR "PROMS") AND ("Information System*" OR "measurement system*" OR "item bank*" OR "itembank*")))) NOT dt=(meeting abstract) AND la=(english OR german OR dutch OR french) AND py=(2007 OR 2008 OR 2009 OR 2010 OR 2011 OR 2012 OR 2013 OR 2014 OR 2015 OR 2016 OR 2017 OR 2018 OR 2019 OR 2020 OR 2021 OR 2022)

*Cochrane Library*

(("cerebrovascular accident" OR "Stroke" OR "strokes" OR "CVA" OR "CVAs" OR "Cerebrovascular Accident" OR "Cerebrovascular Accidents" OR "Cerebrovascular Insult" OR "Cerebrovascular Insults" OR "CVI" OR "CVIs" OR "Brain Attack" OR "Brain Attacks" OR "Brain Infarct*" OR "Brain Infarction" OR "Cerebrovascular Apoplexy" OR "Apoplexy" OR "Brain Vascular Accident" OR "Brain Vascular Accidents" OR "Cerebrovascular Stroke" OR "Cerebrovascular Strokes" OR "Cerebral Stroke" OR "Cerebral Strokes" OR "Acute Stroke" OR "Acute Strokes" OR "Acute Cerebrovascular Accident" OR "Acute Cerebrovascular Accidents" OR "Cerebrovascular Event" OR "Cerebrovascular Events" OR "Cerebrovascular Attack" OR "Cerebrovascular Attacks" OR "Cerebral infarct*" OR "Brain ischemia" OR "Brain ischemia" OR "Brain ischaemia" OR "Cerebral Ischemia" OR "Cerebral Ischaemia" OR "Brain Hemorrhage" OR "Cerebral Hemorrhag*" OR "Cerebral Haemorrhag*" OR "Intracerebral Hemorrhag*" OR "Brain Hemorrhag*" OR "Brain Haemorrhag*" OR "Intracerebral Haemorrhag*" OR "Neurologic Event" OR "Neurologic Events" OR "Transient Ischemic Attack" OR "transient ischemic attack" OR "transient ischemic attack" OR "transient ischaemic attack" OR "transient ischaemic attacks" OR "TIA" OR "TIAs" OR "transient brainstem ischemia" OR "transient brainstem ischaemia" OR "transient brain stem ischemia" OR "transient brain stem ischaemia" OR "transient brain ischemia" OR "transient brain ischaemia" OR "transient cerebral ischemia" OR "transient cerebral ischaemia" OR "transient cerebral ischemic" OR "transient cerebral ischaemic" OR ("transient" AND ("brain" OR "cerebral") AND (ischemi* OR ischaem*))) AND ("PROMIS" OR "PROMIS10" OR "NIHPROMIS" OR "PROMISPI" OR "PROMISPF" OR "PROMISGH" OR "PROMISSF" OR "Patient Reported Outcome Measurement Information System" OR "patient reported outcome measurement system" OR "Patient Reported Outcomes Measurement Information System" OR "patient reported outcomes measurement system")):ti,ab,kw NOT (conference abstract):pt

*Emcare*

((exp "cerebrovascular accident"/ OR "Stroke".mp OR "strokes".mp OR "CVA".mp OR "CVAs".mp OR "Cerebrovascular Accident".mp OR "Cerebrovascular Accidents".mp OR "Cerebrovascular Insult".mp OR "Cerebrovascular Insults".mp OR "CVI".mp OR "CVIs".mp OR "Brain Attack".mp OR "Brain Attacks".mp OR "Brain Infarct*".mp OR exp "Brain Infarction"/ OR "Cerebrovascular Apoplexy".mp OR "Apoplexy".mp OR "Brain Vascular Accident".mp OR "Brain Vascular Accidents".mp OR "Cerebrovascular Stroke".mp OR "Cerebrovascular Strokes".mp OR "Cerebral Stroke".mp OR "Cerebral Strokes".mp OR "Acute Stroke".mp OR "Acute Strokes".mp OR "Acute Cerebrovascular Accident".mp OR "Acute Cerebrovascular Accidents".mp OR "Cerebrovascular Event".mp OR "Cerebrovascular Events".mp OR "Cerebrovascular Attack".mp OR "Cerebrovascular Attacks".mp OR "Cerebral infarct*".mp OR exp "Brain ischemia"/ OR "Brain ischemia".mp OR "Brain ischaemia".mp OR "Cerebral Ischemia".mp OR "Cerebral Ischaemia".mp OR exp "Brain Hemorrhage"/ OR "Cerebral Hemorrhag*".mp OR "Cerebral Haemorrhag*".mp OR "Intracerebral Hemorrhag*".mp OR "Brain Hemorrhag*".mp OR "Brain Haemorrhag*".mp OR "Intracerebral Haemorrhag*".mp OR "Neurologic Event".mp OR "Neurologic Events".mp OR "Transient Ischemic Attack"/ OR "transient ischemic attack".mp OR "transient ischemic attack".mp OR "transient ischaemic attack".mp OR "transient ischaemic attacks".mp OR "TIA".mp OR "TIAs".mp OR "transient brainstem ischemia".mp OR "transient brainstem ischaemia".mp OR "transient brain stem ischemia".mp OR "transient brain stem ischaemia".mp OR "transient brain ischemia".mp OR "transient brain ischaemia".mp OR "transient cerebral ischemia".mp OR "transient cerebral ischaemia".mp OR "transient cerebral ischemic".mp OR "transient cerebral ischaemic".mp OR ("transient".mp ADJ3 ("brain".mp OR "cerebral".mp) ADJ3 (ischemi*.mp OR ischaem*.mp))) AND ("PROMIS".mp OR "PROMIS10".mp OR "NIHPROMIS".mp OR "PROMISPI".mp OR "PROMISPF".mp OR "PROMISGH".mp OR "PROMISSF".mp OR "Patient Reported Outcome Measurement Information System".mp OR "patient reported outcome measurement system".mp OR "patient reported outcome measurement".mp OR "Patient Reported Outcomes Measurement Information System".mp OR "patient reported outcomes measurement system".mp OR "patient reported outcomes measurement".mp OR (("Patient-Reported Outcome*".mp OR "Patientreported Outcome*".mp OR "PROM".mp OR "PROMS".mp) AND ("Information System*".mp OR "measurement system*".mp OR "item bank*".mp OR "itembank*".mp)))) AND (english.la OR german.la OR dutch.la OR french.la) AND (2007 OR 2008 OR 2009 OR 2010 OR 2011 OR 2012 OR 2013 OR 2014 OR 2015 OR 2016 OR 2017 OR 2018 OR 2019 OR 2020 OR 2021 OR 2022).yr

*PsycINFO*

TX(("cerebrovascular accident" OR "Stroke" OR "strokes" OR "CVA" OR "CVAs" OR "Cerebrovascular Accident" OR "Cerebrovascular Accidents" OR "Cerebrovascular Insult" OR "Cerebrovascular Insults" OR "CVI" OR "CVIs" OR "Brain Attack" OR "Brain Attacks" OR "Brain Infarct*" OR "Brain Infarction" OR "Cerebrovascular Apoplexy" OR "Apoplexy" OR "Brain Vascular Accident" OR "Brain Vascular Accidents" OR "Cerebrovascular Stroke" OR "Cerebrovascular Strokes" OR "Cerebral Stroke" OR "Cerebral Strokes" OR "Acute Stroke" OR "Acute Strokes" OR "Acute Cerebrovascular Accident" OR "Acute Cerebrovascular Accidents" OR "Cerebrovascular Event" OR "Cerebrovascular Events" OR "Cerebrovascular Attack" OR "Cerebrovascular Attacks" OR "Cerebral infarct*" OR "Brain ischemia" OR "Brain ischemia" OR "Brain ischaemia" OR "Cerebral Ischemia" OR "Cerebral Ischaemia" OR "Brain Hemorrhage" OR "Cerebral Hemorrhag*" OR "Cerebral Haemorrhag*" OR "Intracerebral Hemorrhag*" OR "Brain Hemorrhag*" OR "Brain Haemorrhag*" OR "Intracerebral Haemorrhag*" OR "Neurologic Event" OR "Neurologic Events" OR "Transient Ischemic Attack" OR "transient ischemic attack" OR "transient ischemic attack" OR "transient ischaemic attack" OR "transient ischaemic attacks" OR "TIA" OR "TIAs" OR "transient brainstem ischemia" OR "transient brainstem ischaemia" OR "transient brain stem ischemia" OR "transient brain stem ischaemia" OR "transient brain ischemia" OR "transient brain ischaemia" OR "transient cerebral ischemia" OR "transient cerebral ischaemia" OR "transient cerebral ischemic" OR "transient cerebral ischaemic" OR ("transient" AND ("brain" OR "cerebral") AND (ischemi* OR ischaem*))) AND ("PROMIS" OR "PROMIS10" OR "NIHPROMIS" OR "PROMISPI" OR "PROMISPF" OR "PROMISGH" OR "PROMISSF" OR "Patient Reported Outcome Measurement Information System" OR "patient reported outcome measurement system" OR "patient reported outcome measurement" OR "Patient Reported Outcomes Measurement Information System" OR "patient reported outcomes measurement system" OR "patient reported outcomes measurement" OR (("Patient-Reported Outcome*" OR "Patientreported Outcome*" OR "PROM" OR "PROMS") AND ("Information System*" OR "measurement system*" OR "item bank*" OR "itembank*")))) AND (english.la OR german.la OR dutch.la OR french.la) AND (2007 OR 2008 OR 2009 OR 2010 OR 2011 OR 2012 OR 2013 OR 2014 OR 2015 OR 2016 OR 2017 OR 2018 OR 2019 OR 2020 OR 20211 OR 2022).yr

*Academic Search Premier*

(TI("cerebrovascular accident" OR "Stroke" OR "strokes" OR "CVA" OR "CVAs" OR "Cerebrovascular Accident" OR "Cerebrovascular Accidents" OR "Cerebrovascular Insult" OR "Cerebrovascular Insults" OR "CVI" OR "CVIs" OR "Brain Attack" OR "Brain Attacks" OR "Brain Infarct*" OR "Brain Infarction" OR "Cerebrovascular Apoplexy" OR "Apoplexy" OR "Brain Vascular Accident" OR "Brain Vascular Accidents" OR "Cerebrovascular Stroke" OR "Cerebrovascular Strokes" OR "Cerebral Stroke" OR "Cerebral Strokes" OR "Acute Stroke" OR "Acute Strokes" OR "Acute Cerebrovascular Accident" OR "Acute Cerebrovascular Accidents" OR "Cerebrovascular Event" OR "Cerebrovascular Events" OR "Cerebrovascular Attack" OR "Cerebrovascular Attacks" OR "Cerebral infarct*" OR "Brain ischemia" OR "Brain ischemia" OR "Brain ischaemia" OR "Cerebral Ischemia" OR "Cerebral Ischaemia" OR "Brain Hemorrhage" OR "Cerebral Hemorrhag*" OR "Cerebral Haemorrhag*" OR "Intracerebral Hemorrhag*" OR "Brain Hemorrhag*" OR "Brain Haemorrhag*" OR "Intracerebral Haemorrhag*" OR "Neurologic Event" OR "Neurologic Events" OR "Transient Ischemic Attack" OR "transient ischemic attack" OR "transient ischemic attack" OR "transient ischaemic attack" OR "transient ischaemic attacks" OR "TIA" OR "TIAs" OR "transient brainstem ischemia" OR "transient brainstem ischaemia" OR "transient brain stem ischemia" OR "transient brain stem ischaemia" OR "transient brain ischemia" OR "transient brain ischaemia" OR "transient cerebral ischemia" OR "transient cerebral ischaemia" OR "transient cerebral ischemic" OR "transient cerebral ischaemic" OR ("transient" AND ("brain" OR "cerebral") AND (ischemi* OR ischaem*))) AND TX("PROMIS" OR "PROMIS10" OR "NIHPROMIS" OR "PROMISPI" OR "PROMISPF" OR "PROMISGH" OR "PROMISSF" OR "Patient Reported Outcome Measurement Information System" OR "patient reported outcome measurement system" OR "Patient Reported Outcomes Measurement Information System" OR "patient reported outcomes measurement system")) **OR** (TX("cerebrovascular accident" OR "Stroke" OR "strokes" OR "CVA" OR "CVAs" OR "Cerebrovascular Accident" OR "Cerebrovascular Accidents" OR "Cerebrovascular Insult" OR "Cerebrovascular Insults" OR "CVI" OR "CVIs" OR "Brain Attack" OR "Brain Attacks" OR "Brain Infarct*" OR "Brain Infarction" OR "Cerebrovascular Apoplexy" OR "Apoplexy" OR "Brain Vascular Accident" OR "Brain Vascular Accidents" OR "Cerebrovascular Stroke" OR "Cerebrovascular Strokes" OR "Cerebral Stroke" OR "Cerebral Strokes" OR "Acute Stroke" OR "Acute Strokes" OR "Acute Cerebrovascular Accident" OR "Acute Cerebrovascular Accidents" OR "Cerebrovascular Event" OR "Cerebrovascular Events" OR "Cerebrovascular Attack" OR "Cerebrovascular Attacks" OR "Cerebral infarct*" OR "Brain ischemia" OR "Brain ischemia" OR "Brain ischaemia" OR "Cerebral Ischemia" OR "Cerebral Ischaemia" OR "Brain Hemorrhage" OR "Cerebral Hemorrhag*" OR "Cerebral Haemorrhag*" OR "Intracerebral Hemorrhag*" OR "Brain Hemorrhag*" OR "Brain Haemorrhag*" OR "Intracerebral Haemorrhag*" OR "Neurologic Event" OR "Neurologic Events" OR "Transient Ischemic Attack" OR "transient ischemic attack" OR "transient ischemic attack" OR "transient ischaemic attack" OR "transient ischaemic attacks" OR "TIA" OR "TIAs" OR "transient brainstem ischemia" OR "transient brainstem ischaemia" OR "transient brain stem ischemia" OR "transient brain stem ischaemia" OR "transient brain ischemia" OR "transient brain ischaemia" OR "transient cerebral ischemia" OR "transient cerebral ischaemia" OR "transient cerebral ischemic" OR "transient cerebral ischaemic" OR ("transient" AND ("brain" OR "cerebral") AND (ischemi* OR ischaem*))) AND TI("PROMIS" OR "PROMIS10" OR "NIHPROMIS" OR "PROMISPI" OR "PROMISPF" OR "PROMISGH" OR "PROMISSF" OR "Patient Reported Outcome Measurement Information System" OR "patient reported outcome measurement system" OR "Patient Reported Outcomes Measurement Information System" OR "patient reported outcomes measurement system")) **OR** TI(("cerebrovascular accident" OR "Stroke" OR "strokes" OR "CVA" OR "CVAs" OR "Cerebrovascular Accident" OR "Cerebrovascular Accidents" OR "Cerebrovascular Insult" OR "Cerebrovascular Insults" OR "CVI" OR "CVIs" OR "Brain Attack" OR "Brain Attacks" OR "Brain Infarct*" OR "Brain Infarction" OR "Cerebrovascular Apoplexy" OR "Apoplexy" OR "Brain Vascular Accident" OR "Brain Vascular Accidents" OR "Cerebrovascular Stroke" OR "Cerebrovascular Strokes" OR "Cerebral Stroke" OR "Cerebral Strokes" OR "Acute Stroke" OR "Acute Strokes" OR "Acute Cerebrovascular Accident" OR "Acute Cerebrovascular Accidents" OR "Cerebrovascular Event" OR "Cerebrovascular Events" OR "Cerebrovascular Attack" OR "Cerebrovascular Attacks" OR "Cerebral infarct*" OR "Brain ischemia" OR "Brain ischemia" OR "Brain ischaemia" OR "Cerebral Ischemia" OR "Cerebral Ischaemia" OR "Brain Hemorrhage" OR "Cerebral Hemorrhag*" OR "Cerebral Haemorrhag*" OR "Intracerebral Hemorrhag*" OR "Brain Hemorrhag*" OR "Brain Haemorrhag*" OR "Intracerebral Haemorrhag*" OR "Neurologic Event" OR "Neurologic Events" OR "Transient Ischemic Attack" OR "transient ischemic attack" OR "transient ischemic attack" OR "transient ischaemic attack" OR "transient ischaemic attacks" OR "TIA" OR "TIAs" OR "transient brainstem ischemia" OR "transient brainstem ischaemia" OR "transient brain stem ischemia" OR "transient brain stem ischaemia" OR "transient brain ischemia" OR "transient brain ischaemia" OR "transient cerebral ischemia" OR "transient cerebral ischaemia" OR "transient cerebral ischemic" OR "transient cerebral ischaemic" OR ("transient" AND ("brain" OR "cerebral") AND (ischemi* OR ischaem*))) AND ("PROMIS" OR "PROMIS10" OR "NIHPROMIS" OR "PROMISPI" OR "PROMISPF" OR "PROMISGH" OR "PROMISSF" OR "Patient Reported Outcome Measurement Information System" OR "patient reported outcome measurement system" OR "patient reported outcome measurement" OR "Patient Reported Outcomes Measurement Information System" OR "patient reported outcomes measurement system" OR "patient reported outcomes measurement" OR (("Patient-Reported Outcome*" OR "Patientreported Outcome*" OR "PROM" OR "PROMS") AND ("Information System*" OR "measurement system*" OR "item bank*" OR "itembank*")))) **OR** U(("cerebrovascular accident" OR "Stroke" OR "strokes" OR "CVA" OR "CVAs" OR "Cerebrovascular Accident" OR "Cerebrovascular Accidents" OR "Cerebrovascular Insult" OR "Cerebrovascular Insults" OR "CVI" OR "CVIs" OR "Brain Attack" OR "Brain Attacks" OR "Brain Infarct*" OR "Brain Infarction" OR "Cerebrovascular Apoplexy" OR "Apoplexy" OR "Brain Vascular Accident" OR "Brain Vascular Accidents" OR "Cerebrovascular Stroke" OR "Cerebrovascular Strokes" OR "Cerebral Stroke" OR "Cerebral Strokes" OR "Acute Stroke" OR "Acute Strokes" OR "Acute Cerebrovascular Accident" OR "Acute Cerebrovascular Accidents" OR "Cerebrovascular Event" OR "Cerebrovascular Events" OR "Cerebrovascular Attack" OR "Cerebrovascular Attacks" OR "Cerebral infarct*" OR "Brain ischemia" OR "Brain ischemia" OR "Brain ischaemia" OR "Cerebral Ischemia" OR "Cerebral Ischaemia" OR "Brain Hemorrhage" OR "Cerebral Hemorrhag*" OR "Cerebral Haemorrhag*" OR "Intracerebral Hemorrhag*" OR "Brain Hemorrhag*" OR "Brain Haemorrhag*" OR "Intracerebral Haemorrhag*" OR "Neurologic Event" OR "Neurologic Events" OR "Transient Ischemic Attack" OR "transient ischemic attack" OR "transient ischemic attack" OR "transient ischaemic attack" OR "transient ischaemic attacks" OR "TIA" OR "TIAs" OR "transient brainstem ischemia" OR "transient brainstem ischaemia" OR "transient brain stem ischemia" OR "transient brain stem ischaemia" OR "transient brain ischemia" OR "transient brain ischaemia" OR "transient cerebral ischemia" OR "transient cerebral ischaemia" OR "transient cerebral ischemic" OR "transient cerebral ischaemic" OR ("transient" AND ("brain" OR "cerebral") AND (ischemi* OR ischaem*))) AND ("PROMIS" OR "PROMIS10" OR "NIHPROMIS" OR "PROMISPI" OR "PROMISPF" OR "PROMISGH" OR "PROMISSF" OR "Patient Reported Outcome Measurement Information System" OR "patient reported outcome measurement system" OR "patient reported outcome measurement" OR "Patient Reported Outcomes Measurement Information System" OR "patient reported outcomes measurement system" OR "patient reported outcomes measurement" OR (("Patient-Reported Outcome*" OR "Patientreported Outcome*" OR "PROM" OR "PROMS") AND ("Information System*" OR "measurement system*" OR "item bank*" OR "itembank*")))) **OR** KW(("cerebrovascular accident" OR "Stroke" OR "strokes" OR "CVA" OR "CVAs" OR "Cerebrovascular Accident" OR "Cerebrovascular Accidents" OR "Cerebrovascular Insult" OR "Cerebrovascular Insults" OR "CVI" OR "CVIs" OR "Brain Attack" OR "Brain Attacks" OR "Brain Infarct*" OR "Brain Infarction" OR "Cerebrovascular Apoplexy" OR "Apoplexy" OR "Brain Vascular Accident" OR "Brain Vascular Accidents" OR "Cerebrovascular Stroke" OR "Cerebrovascular Strokes" OR "Cerebral Stroke" OR "Cerebral Strokes" OR "Acute Stroke" OR "Acute Strokes" OR "Acute Cerebrovascular Accident" OR "Acute Cerebrovascular Accidents" OR "Cerebrovascular Event" OR "Cerebrovascular Events" OR "Cerebrovascular Attack" OR "Cerebrovascular Attacks" OR "Cerebral infarct*" OR "Brain ischemia" OR "Brain ischemia" OR "Brain ischaemia" OR "Cerebral Ischemia" OR "Cerebral Ischaemia" OR "Brain Hemorrhage" OR "Cerebral Hemorrhag*" OR "Cerebral Haemorrhag*" OR "Intracerebral Hemorrhag*" OR "Brain Hemorrhag*" OR "Brain Haemorrhag*" OR "Intracerebral Haemorrhag*" OR "Neurologic Event" OR "Neurologic Events" OR "Transient Ischemic Attack" OR "transient ischemic attack" OR "transient ischemic attack" OR "transient ischaemic attack" OR "transient ischaemic attacks" OR "TIA" OR "TIAs" OR "transient brainstem ischemia" OR "transient brainstem ischaemia" OR "transient brain stem ischemia" OR "transient brain stem ischaemia" OR "transient brain ischemia" OR "transient brain ischaemia" OR "transient cerebral ischemia" OR "transient cerebral ischaemia" OR "transient cerebral ischemic" OR "transient cerebral ischaemic" OR ("transient" AND ("brain" OR "cerebral") AND (ischemi* OR ischaem*))) AND ("PROMIS" OR "PROMIS10" OR "NIHPROMIS" OR "PROMISPI" OR "PROMISPF" OR "PROMISGH" OR "PROMISSF" OR "Patient Reported Outcome Measurement Information System" OR "patient reported outcome measurement system" OR "patient reported outcome measurement" OR "Patient Reported Outcomes Measurement Information System" OR "patient reported outcomes measurement system" OR "patient reported outcomes measurement" OR (("Patient-Reported Outcome*" OR "Patientreported Outcome*" OR "PROM" OR "PROMS") AND ("Information System*" OR "measurement system*" OR "item bank*" OR "itembank*")))) **OR** AB(("cerebrovascular accident" OR "Stroke" OR "strokes" OR "CVA" OR "CVAs" OR "Cerebrovascular Accident" OR "Cerebrovascular Accidents" OR "Cerebrovascular Insult" OR "Cerebrovascular Insults" OR "CVI" OR "CVIs" OR "Brain Attack" OR "Brain Attacks" OR "Brain Infarct*" OR "Brain Infarction" OR "Cerebrovascular Apoplexy" OR "Apoplexy" OR "Brain Vascular Accident" OR "Brain Vascular Accidents" OR "Cerebrovascular Stroke" OR "Cerebrovascular Strokes" OR "Cerebral Stroke" OR "Cerebral Strokes" OR "Acute Stroke" OR "Acute Strokes" OR "Acute Cerebrovascular Accident" OR "Acute Cerebrovascular Accidents" OR "Cerebrovascular Event" OR "Cerebrovascular Events" OR "Cerebrovascular Attack" OR "Cerebrovascular Attacks" OR "Cerebral infarct*" OR "Brain ischemia" OR "Brain ischemia" OR "Brain ischaemia" OR "Cerebral Ischemia" OR "Cerebral Ischaemia" OR "Brain Hemorrhage" OR "Cerebral Hemorrhag*" OR "Cerebral Haemorrhag*" OR "Intracerebral Hemorrhag*" OR "Brain Hemorrhag*" OR "Brain Haemorrhag*" OR "Intracerebral Haemorrhag*" OR "Neurologic Event" OR "Neurologic Events" OR "Transient Ischemic Attack" OR "transient ischemic attack" OR "transient ischemic attack" OR "transient ischaemic attack" OR "transient ischaemic attacks" OR "TIA" OR "TIAs" OR "transient brainstem ischemia" OR "transient brainstem ischaemia" OR "transient brain stem ischemia" OR "transient brain stem ischaemia" OR "transient brain ischemia" OR "transient brain ischaemia" OR "transient cerebral ischemia" OR "transient cerebral ischaemia" OR "transient cerebral ischemic" OR "transient cerebral ischaemic" OR ("transient" AND ("brain" OR "cerebral") AND (ischemi* OR ischaem*))) AND ("PROMIS" OR "PROMIS10" OR "NIHPROMIS" OR "PROMISPI" OR "PROMISPF" OR "PROMISGH" OR "PROMISSF" OR "Patient Reported Outcome Measurement Information System" OR "patient reported outcome measurement system" OR "patient reported outcome measurement" OR "Patient Reported Outcomes Measurement Information System" OR "patient reported outcomes measurement system" OR "patient reported outcomes measurement" OR (("Patient-Reported Outcome*" OR "Patientreported Outcome*" OR "PROM" OR "PROMS") AND ("Information System*" OR "measurement system*" OR "item bank*" OR "itembank*")))) AND (english.la OR german.la OR dutch.la OR french.la) AND (2007 OR 2008 OR 2009 OR 2010 OR 2011 OR 2012 OR 2013 OR 2014 OR 2015 OR 2016 OR 2017 OR 2018 OR 2019 OR 2020 OR 2021 OR 2022).yr

*Google Scholar*

Search A.

"cerebrovascular accident"|"Stroke"|"CVA" "PROMIS"|"Patient Reported Outcome Measurement Information System"|"patient reported outcome measurement system"|"Patient Reported Outcomes Measurement Information System"|"Patient Reported Outcomes Measurement Information System"

Search B.

"cerebrovascular accident"|"Stroke"|"CVA" "PROMIS"

Search C in title.

"cerebrovascular accident"|"Stroke"|"CVA" "PROMIS"|"Patient Reported Outcome Measurement Information System"|"patient reported outcome measurement system"|"Patient Reported Outcomes Measurement Information System"|"Patient Reported Outcomes Measurement Information System"

Search D in title.

"cerebrovascular accident"|"Stroke"|"CVA" "PROMIS"
